# Assessing the impact of comorbid type 2 diabetes mellitus on the disease burden of chronic hepatitis B virus infection and its complications in China from 2006 to 2030: a modeling study

**DOI:** 10.1186/s41256-024-00345-2

**Published:** 2024-01-22

**Authors:** Jinzhao Xie, Xu Wang, Xinran Wang, Jinghua Li, Yusheng Jie, Yuantao Hao, Jing Gu

**Affiliations:** 1 Department of Medical Statistics, School of Public Health, Sun Yat-sen University, Guangzhou, China; 2 Sun Yat-sen Global Health Institute, School of Public Health and Institute of State Governance, Sun Yat-sen University, Guangzhou, China; 3 Key Laboratory of Health Informatics of Guangdong Province, Sun Yat-sen University, Guangzhou, China; 4 Department of Infectious Diseases, The Third Affiliated Hospital, Sun Yat-sen University, Guangzhou, China; 5 Center for Public Health and Epidemic Preparedness and Response, Peking University, Beijing, China

**Keywords:** Hepatitis B virus infection, Diabetes mellitus, Comorbidity, China, Markov, Disease burden

## Abstract

**Background:**

China bears a high burden of both hepatitis B virus (HBV) infection and type 2 diabetes mellitus (T2DM). T2DM accelerates the progression of liver disease among individuals infected with HBV. This study aims to assess the excess disease burden caused by comorbid T2DM among HBV-infected individuals in China.

**Methods:**

We estimated the disease burden of HBV and its complications in China from 2006 to 2030 using individual-based Markov models. The baseline population consisted of 93 million HBV-infected individuals derived from the 2006 National Serological Epidemiological Survey. We developed two models: one incorporated the impact of T2DM on the disease progression of HBV infection, while the other did not consider the impact of T2DM. By comparing the outcomes between these two models, we estimated the excess disease burden attributable to comorbid T2DM among HBV-infected individuals.

**Results:**

The incidence of severe HBV complications, including cirrhosis, hepatocellular carcinoma (HCC), and liver-related deaths, exhibited an increasing trend from 2006 to 2030 among the Chinese HBV-infected population. Comorbid T2DM increased the annual incidence and cumulative cases of severe HBV complications. From 2006 to 2022, comorbid T2DM caused 791,000 (11.41%), 244,000 (9.27%), 377,000 (8.78%), and 796,000 (12.19%) excess cases of compensated cirrhosis, decompensated cirrhosis, HCC, and liver-related deaths, respectively. From 2023 to 2030, comorbid T2DM is projected to result in an 8.69% excess in severe HBV complications and an 8.95% increase in liver-related deaths. Among individuals aged 60 and older at baseline, comorbid T2DM led to a 21.68% excess in severe HBV complications and a 28.70% increase in liver-related deaths from 2006 to 2022, with projections indicating a further 20.76% increase in severe HBV complications and an 18.31% rise in liver-related deaths over the next seven years.

**Conclusions:**

Comorbid T2DM imposes a substantial disease burden on individuals with HBV infection in China. Healthcare providers and health policymakers should develop and implement tailored strategies for the effective management and control of T2DM in individuals with HBV infection.

**Supplementary Information:**

The online version contains supplementary material available at 10.1186/s41256-024-00345-2.

## Background

Hepatitis B virus (HBV) infection imposes a substantial burden on global health, resulting in approximately 820,000 annual deaths [1]. The World Health Organization (WHO) has implemented various prevention and treatment strategies to achieve the goal of eliminating HBV by 2030. These strategies include expanding the coverage of the hepatitis B vaccine (HepB) and improving access to long-term suppressive treatment [2, 3]. Despite the progress achieved through the effective HBV prevention program, approximately 300 million individuals are still living with chronic HBV infection [4, 5]. Moreover, this population is rapidly aging, rendering them more vulnerable to comorbid noncommunicable diseases (NCDs) such as type 2 diabetes mellitus (T2DM) [6–8].

T2DM is one of the most common comorbidities among HBV-infected individuals. The prevalence of T2DM among the HBV-infected population in the Western Pacific Region has increased by 161.07% over the last three decades [9]. T2DM was also found to be one of the most common NCDs coexisting with HBV infection in North America [8, 10]. HBV infection can lead to liver damage and persistent inflammatory responses, thereby increasing the risk of developing T2DM [11–13]. Moreover, individuals with T2DM have a high risk of HBV infection due to frequent percutaneous exposure to blood [14, 15]. Therefore, the risk of developing comorbid T2DM is elevated in individuals with HBV due to synergistic interactions between these two diseases. HBV infection follows a complex course, with patients progressing from one phase to another and potentially developing a range of complications, including chronic hepatitis B (CHB), cirrhosis, and hepatocellular carcinoma (HCC) [16]. The presence of comorbid T2DM can accelerate the progression of severe liver complications in HBV-infected individuals [17], which may be due to increased levels of free fatty acids, hepatic oxidative stress, and hyperinsulinemia caused by T2DM [18–20]. Consequently, individuals with HBV infection who also have T2DM are at a higher risk of developing cirrhosis, HCC, liver-related death, and experiencing a worse prognosis compared to those with HBV infection alone [21–24].

Despite remarkable progress in HBV control over the past few decades, China still carries the highest burden of HBV infection worldwide, with 87 million individuals living with chronic HBV infection [7, 25]. Additionally, China also bears a substantial burden of T2DM, with an estimated 141 million affected adults and a prevalence of 12.4% [26]. T2DM is a common comorbidity among Chinese HBV-infected individuals and can lead to a worse disease prognosis among this population [9]. A comprehensive assessment of the disease burden attributed to comorbid T2DM among the HBV-infected population can provide a foundation for the development of tailored health strategies targeting comorbid T2DM. However, none of the previous modeling studies regarding the disease burden of the HBV-infected population in China considered the burden caused by comorbid T2DM, and there is no large cohort study assessing the impact of comorbid T2DM on the long-term outcomes of HBV-infected individuals in China [27–29]. The impact of comorbid T2DM on the disease burden of Chinese HBV-infected population remains unclear.

This study aimed to develop individual-level Markov models to estimate the excess disease burden, including CHB, cirrhosis, decompensated cirrhosis, HCC, and liver-related death, caused by comorbid T2DM among Chinese HBV-infected individuals over the past decades and predict these burdens for the next decade.

## Methods

### Study design

Individual-level, dynamic Markov models were applied to estimate the disease burden of chronic HBV infection in China from 2006 to 2030 and to explore the impact of comorbid T2DM. We developed an individual-level Markov model to incorporate the impact of T2DM on the disease progression of HBV infection, referred to as the HBV-T2DM model, and estimated the disease burden in HBV-infected individuals from 2006 to 2030. The disease burden encompasses the annual and cumulative incident cases of CHB, compensated cirrhosis, decompensated cirrhosis, HCC, and liver-related death. In addition, we created another Markov model with the same structure, referred to as the HBV model, which did not account for the impact of T2DM. By comparing the outcomes between the HBV-T2DM model and the HBV model, we estimated the excess disease burden attributed to comorbid T2DM among HBV-infected individuals. Furthermore, we performed subgroup analyses to estimate the excess disease burden among adults aged 60 years and older and individuals between the ages of 45 and 59 at baseline. The model construction was conducted using TreeAge Pro 2022 R.1.2, and data analyses were performed using the R software package, version 4.1.1.

### Model construction

**Fig. 1:**
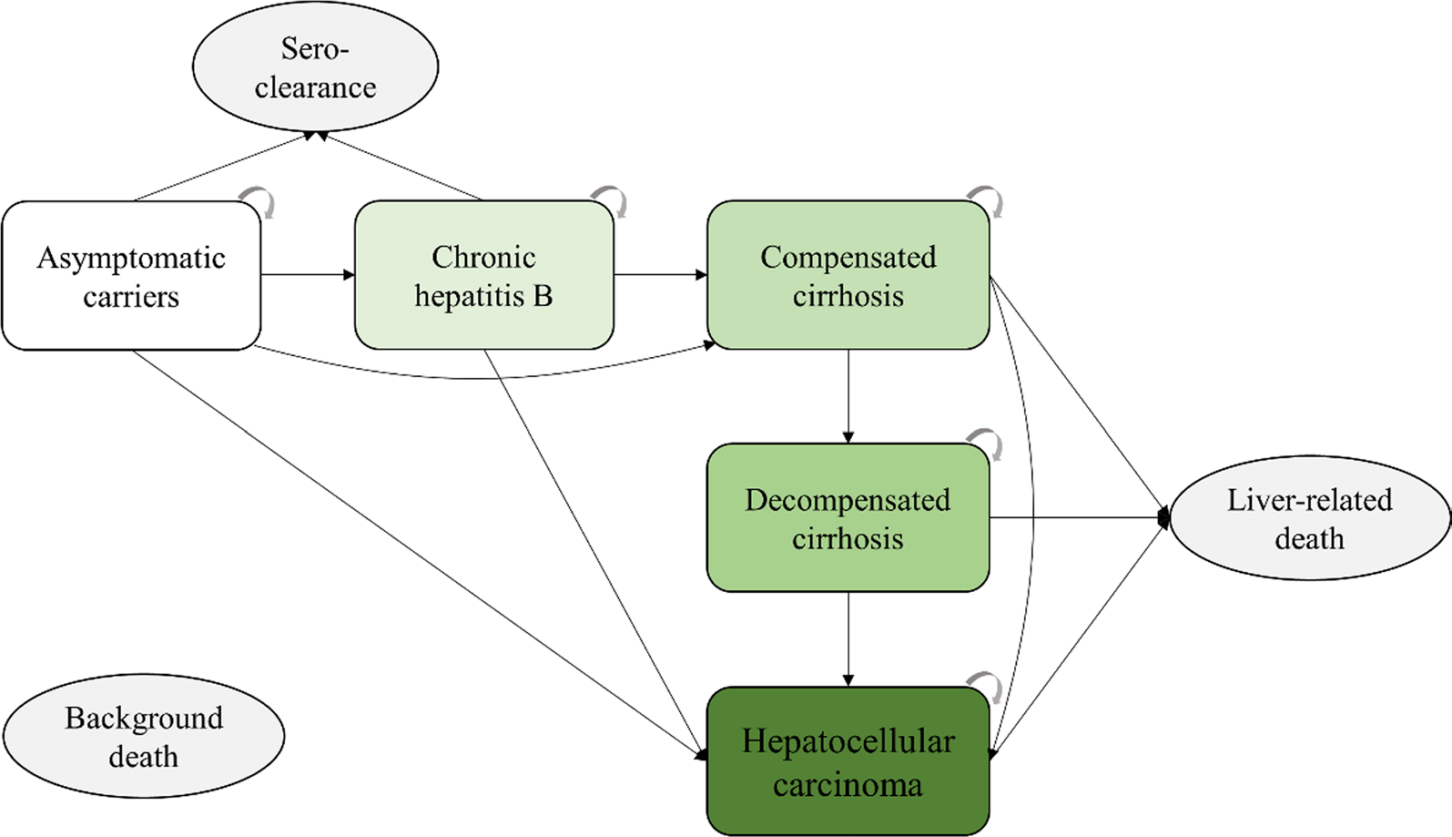
Schematic diagram of the Markov model for disease progression of HBV infection. The arrows represent transitions between states. The color intensity represents disease severity, with darker colors indicating more severe conditions. Seroclearance and death are the absorbing states, with death further classified into liver-related death and background death

Based on the natural history of chronic HBV infection, we developed a Markov model to represent the different health states and disease progression of HBV-infected individuals (Fig. 1) [16]. The model consists of seven health states: seroclearance, asymptomatic carriers, CHB, compensated cirrhosis, decompensated cirrhosis, HCC, and death. Seroclearance and death are the absorbing states, with death further classified into liver-related death and background death. The definitions of each state are available in the Supplemental Methods section of the Supplementary Material. Individuals can transition from one health state to another or remain in the same state at predefined annual transition probability rates. Asymptomatic carriers can progress to CHB, compensated cirrhosis, or achieve hepatitis B surface antigen (HBsAg) seroclearance. CHB patients may progress to compensated cirrhosis or achieve HBsAg seroclearance. Individuals with compensated cirrhosis can further progress to decompensated cirrhosis. Decompensated cirrhosis patients may develop HCC. Asymptomatic carriers, CHB patients, and compensated cirrhosis patients can directly develop HCC. Individuals with compensated cirrhosis, decompensated cirrhosis, and HCC are at risk of liver-related death. All individuals have a background death rate, which was obtained from the China Population and Employment Statistics Yearbook 2022, published by the National Bureau of Statistics of China [30]. The model structure is illustrated in Fig. 1.

The model parameters include annual transition probabilities of health states and population characteristics. The annual transition probabilities for individuals with HBV infection alone were derived from published literature and are presented in Additional file 1: Table S1. Previous studies have indicated that individuals with HBV-T2DM comorbidity can experience a faster disease progression than those with HBV infection alone, and they have reported the corresponding relative risks (RRs) [17, 21–24]. To estimate the transition probabilities for individuals with HBV-T2DM comorbidity, we multiplied the RRs obtained from previous cohort studies or meta-analyses by the transition probabilities for those with HBV infection alone. The estimated transition probabilities for individuals with HBV-T2DM comorbidity are presented in Additional file 1: Table S2. We assumed that treated patients would have a lower rate of disease progression than those who remained untreated. The treatment rate for CHB patients was set at 10% based on the estimation by the WHO [31, 32]. We assumed that 90% of cirrhosis and HCC patients received treatment according to Chinese experts in hepatology. The model utilized a one-year cycle length and projected annual incident cases and cumulative cases for each health state until 2030.

### Population characteristics

We constructed a simulated cohort to represent the nationwide HBV-infected population. The eligibility criteria for this modeling study are HBsAg positive. We included all individuals with HBV infection, covering all disease states of HBV infection, including asymptomatic carriers, CHB, cirrhosis, and HCC. HBV-infected individuals who were not counted or reported in the published data were excluded from our study. The China CDC conducted a nationwide serological survey for HBV in 2006, estimating the prevalence of HBV and the number of cases. From 2006 to 2023, no large-scale nationwide serological survey for HBV has been conducted. Therefore, we used data from the 2006 National Serological Epidemiological Survey as the baseline population, which is consistent with previous HBV-related modeling studies conducted in China [27, 33]. According to the results of this survey, we included a baseline population of 93 million HBV-infected individuals [34, 35]. The distribution of disease states in the baseline population was based on previous literature and is presented in Additional file 1: Table S3. We incorporated the annual reported data for new CHB patients from 2006 to 2021. Data for the years 2006 to 2019 were obtained from the Chinese Center for Disease Control and Prevention (China CDC) (https://www.phsciencedata.cn/Share/en/index.jsp). Data for 2020 and 2021 were obtained from the China Health Statistics Yearbook 2021 and 2022, respectively (http://www.nhc.gov.cn/wjw/index.shtml). For the years beyond 2021, we included the predicted annual number of new CHB patients derived from our previous modeling study with intervention coverage remaining at the 2020 level [33].

In our models, T2DM patients are defined as individuals with self-reported T2DM diagnosed by a healthcare professional or with a fasting plasma glucose level of 126 mg/dL or higher, a 2-h plasma glucose level of 200 mg/dL or higher after a 75-g oral glucose challenge, or a hemoglobin A_1c_ level of 6.5% or higher [26]. T2DM patients include patients with varying disease severity and treatment situations. The age-specific prevalence of T2DM among the HBV-infected population was estimated by multiplying the prevalence of T2DM in the general population by the prevalence ratio of T2DM in the HBV-infected population compared to the general population. According to a large-scale meta-analysis, this prevalence ratio was estimated to be 1.33 (95% CI 1.09 to 1.62) [13]. The prevalence of T2DM in the general population was obtained from a nationally representative survey [26]. The age-specific prevalence of T2DM among the HBV-infected population is presented in Additional file 1: Table S4.

### Model validation

Following the model validation method used in previous studies, we conducted model validation by comparing our model's results to authoritative published data [27, 33]. According to reports from the China CDC and the WHO, it was estimated that there were 87 million individuals infected with HBV in China in 2016, with 28 million of them having CHB [25]. We compared the number of HBV cases obtained from our model to the figures reported in authoritative sources. Additionally, we compared the number of incident cases of HCC obtained from our model to the annual HCC cases reported by the China National Cancer Center. The results of the model validation are presented in Additional file 1: Table S5.

### Sensitivity analysis

We conducted one-way sensitivity analyses to assess the impact of parameter uncertainties and evaluate the robustness of the model. Specifically, we examined the effects of varying disease progression rates among individuals with HBV-T2DM comorbidity, using both upper and lower range values, on the excess disease burden attributable to comorbid T2DM among the HBV-infected population. The results of the sensitivity analyses are presented in Additional file 1: Figs. S1–S4.

## Results

### Disease burden of HBV and its complications in China

**Fig. 2:**
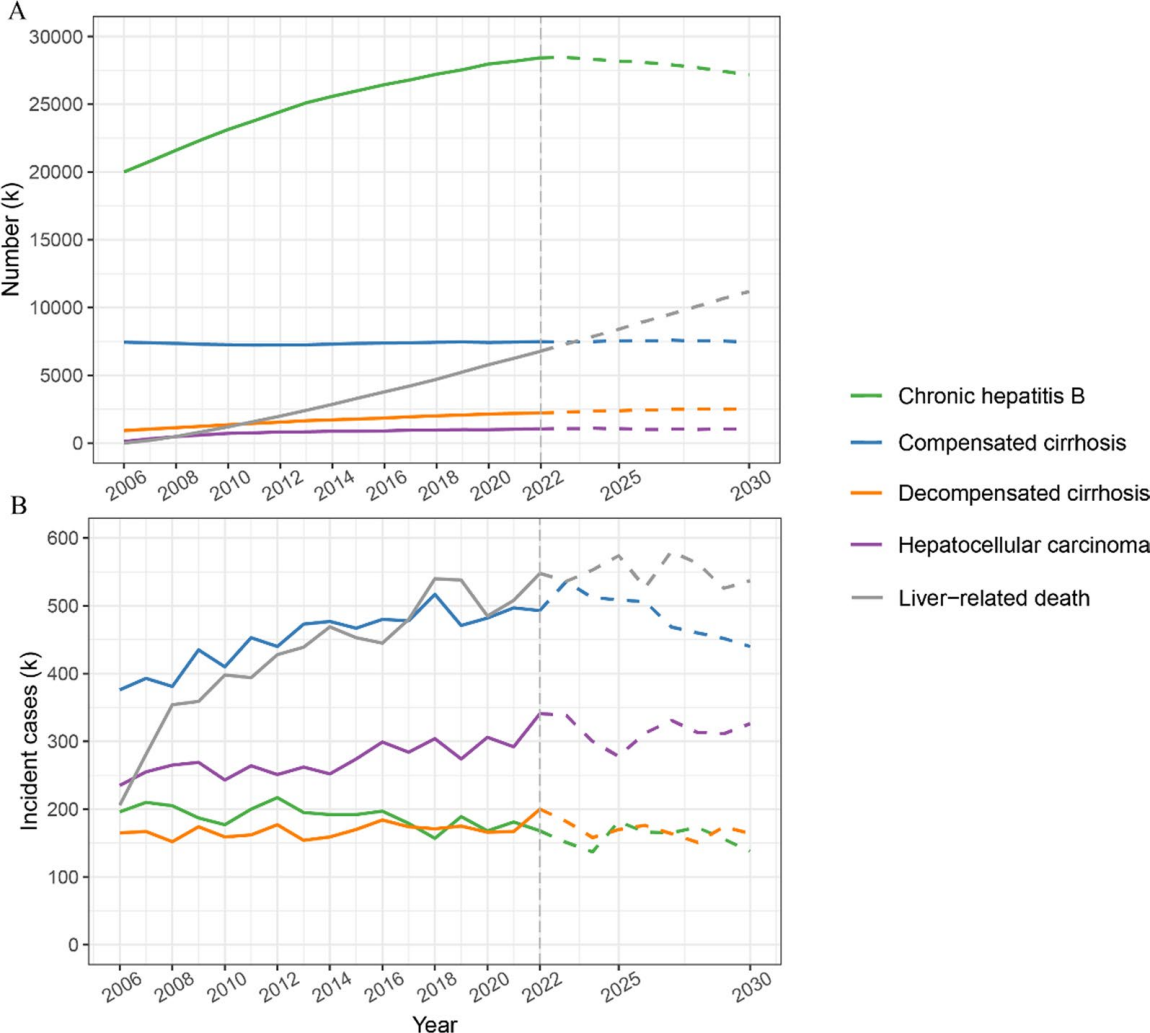
**A** The total number (thousands) of HBV-infected individuals categorized by different disease states from 2006 to 2030. **B** Annual incident cases (thousands) of HBV-infected individuals categorized by different disease states from 2006 to 2030

Table 1 and Fig. 2A present the number of HBV-infected individuals categorized by disease states from 2006 to 2030. During this period, the total number of HBV patients decreased by 20.04%, declining from 93.00 million in 2006 to 84.11 million in 2022, with a projected decrease to 74.36 million in 2030. The number of patients with CHB increased from 20.00 million in 2006 to 28.43 million in 2022 and is projected to decrease to 27.14 million in 2030. Severe HBV complications exhibited an increasing trend from 2006 to 2030. The number of patients with decompensated cirrhosis and HCC increased from 0.92 million and 0.12 million in 2006 to 2.23 million and 1.05 million in 2022 and is projected to reach 2.52 million and 1.07 million in 2030, respectively. The cumulative number of liver-related deaths was 6.78 million in 2022 and is projected to reach 11.18 million in 2030.

**Table 1:** The number of HBV-infected individuals categorized by different disease states (thousands)

Disease states	2006	2010	2022	2025	2030
Asymptomatic carriers	64,506	59,244	44,914	41,701	36,150
CHB	20,004	23,139	28,426	28,179	27,142
Compensated cirrhosis	7449	7259	7492	7534	7485
Decompensated cirrhosis	921	1355	2230	2404	2518
HCC	120	721	1051	1060	1067
Total HBV patients	93,000	91,718	84,113	80,878	74,362
Liver-related death	0	1200	6777	8414	11,184

*CHB* Chronic hepatitis B, *HBV* Hepatitis B virus, *HCC* Hepatocellular carcinoma

Figure 2B illustrates the annual incident cases of HBV complications and liver-related deaths from 2006 to 2030. The incident cases of CHB showed a decreasing trend from 2006 to 2030, decreasing from 196,000 in 2006 to 138,000 in 2030. The incident cases of compensated cirrhosis showed a trend of initially increasing and then decreasing, rising from 376,000 in 2006 to 493,000 in 2022 and decreasing to 440,000 in 2030. The annual incident cases of decompensated cirrhosis remained stable from 2006 to 2030. The incident cases of HCC showed an upward trend, rising from 235,000 in 2006 to 326,000 in 2030. Furthermore, the incident cases of liver-related deaths increased by 163.05% during the same period, rising from 206,000 cases in 2006 to 537,000 cases in 2030.

### Excess burden of HBV complications due to comorbid T2DM among the HBV-infected population

**Fig. 3:**
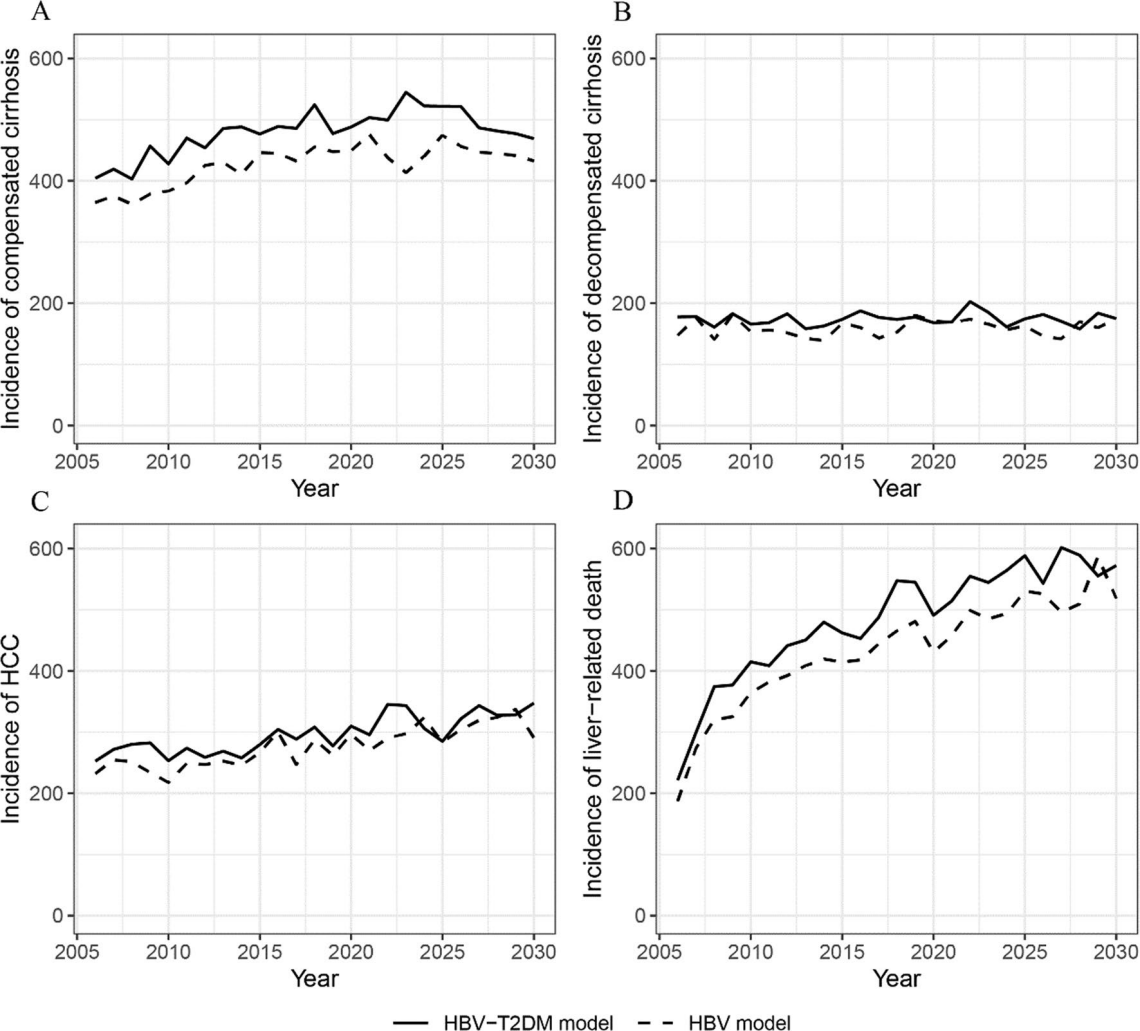
Annual incidence (per 100,000 persons) of severe HBV complications estimated by the HBV model and the HBV-T2DM model. **A** Compensated cirrhosis; **B** decompensated cirrhosis; **C** HCC; **D** liver-related death. *T2DM* Type 2 diabetes mellitus, *HBV* Hepatitis B virus, *HCC* Hepatocellular carcinoma

The annual incidence of HBV complications and liver-related death, as estimated by the HBV model and the HBV-T2DM model, is presented in Fig. 3. The differences between the results obtained from the HBV model and the HBV-T2DM model represent the excess burden of HBV complications and liver-related death attributed to comorbid T2DM among the HBV-infected population. From 2006 to 2030, the HBV-T2DM model showed a higher incidence of compensated cirrhosis, decompensated cirrhosis, HCC, and liver-related death than the HBV model. These results indicate that T2DM increases the incidence of HBV complications and death among HBV-infected individuals. For instance, in 2022, the incidence of compensated cirrhosis and HCC is 437.6 and 290.7 per 100,000 persons, respectively, according to the HBV model. However, based on the HBV-T2DM model, these rates increase to 499.3 and 345.4 per 100,000 persons, respectively. The incidence of liver-related death is 498.9 per 100,000 persons in 2022 according to the HBV model, whereas it is 555.0 per 100,000 persons according to the HBV-T2DM model, indicating an 11.24% excess incidence of liver-related death due to comorbid T2DM among HBV-infected individuals.

**Fig. 4:**
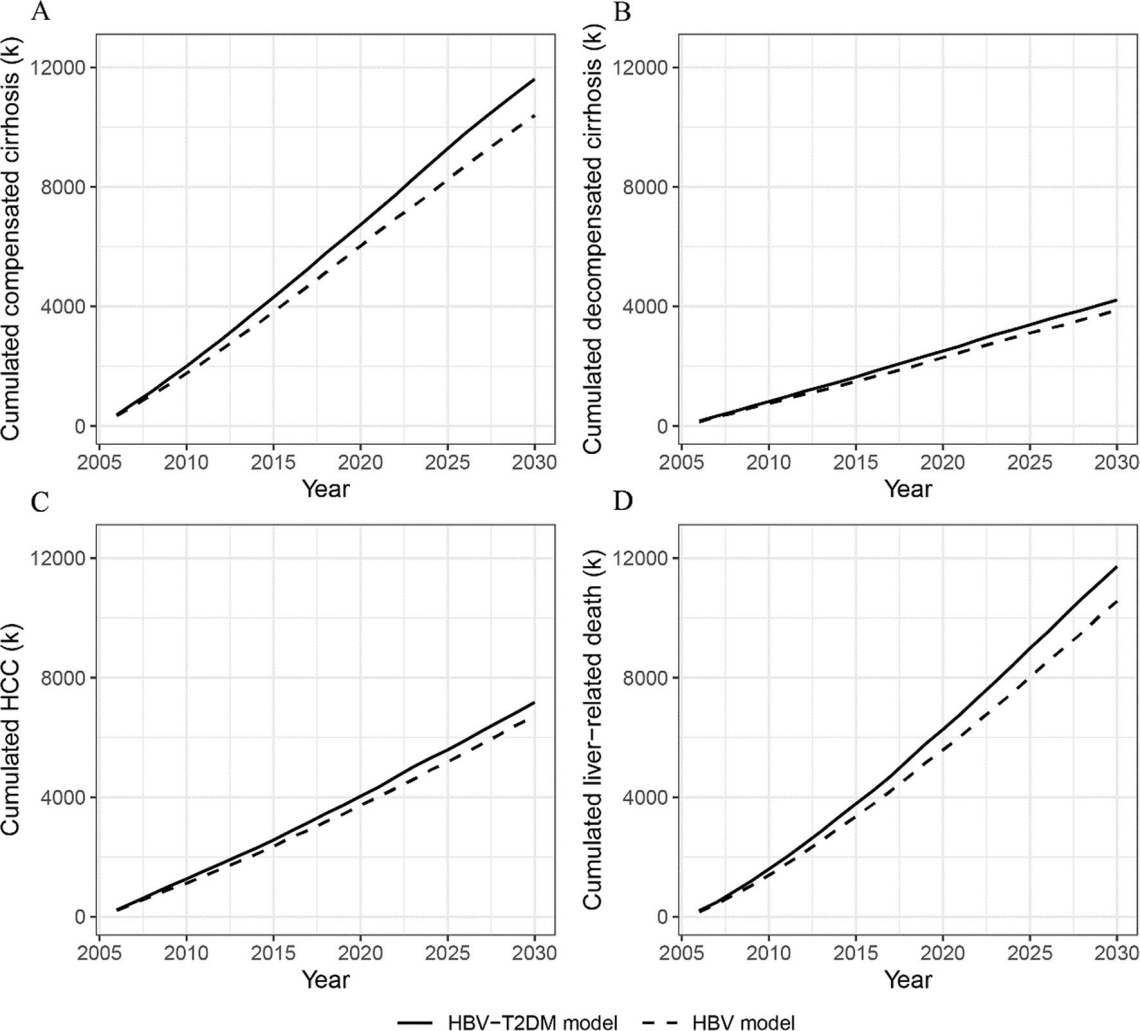
Cumulative incident cases (thousands) of severe HBV complications estimated by the HBV model and the HBV-T2DM model. **A** Compensated cirrhosis; **B** decompensated cirrhosis; **C** HCC; **D** liver-related death. *T2DM* Type 2 diabetes mellitus, *HBV* Hepatitis B virus, *HCC* Hepatocellular carcinoma

Figure 4 illustrates the cumulative incident cases of HBV complications and liver-related death as estimated by the HBV model and HBV-T2DM model. The differences between the results obtained from the two models indicate the excess cumulative incident cases attributed to comorbid T2DM among HBV-infected individuals. The HBV-T2DM model consistently shows higher cumulative incident cases of HBV complications and liver-related death compared to the HBV model from 2006 to 2030. Moreover, the excess cumulative incident cases of compensated cirrhosis, decompensated cirrhosis, HCC, and liver-related deaths caused by comorbid T2DM exhibit an increasing trend from 2006 to 2030 (Fig. 4). Table 2 presents the excess burden attributable to comorbid T2DM among the Chinese HBV-infected population. From 2006 to 2022, comorbid T2DM resulted in an additional 1.41 million cases (10.19%) of severe HBV complications among the Chinese HBV-infected population. Comorbid T2DM caused an additional 791,000 cases (11.41%), 244,000 cases (9.27%), and 377,000 cases (8.78%) of compensated cirrhosis, decompensated cirrhosis, and HCC, respectively. Additionally, comorbid T2DM led to 796,000 excess liver-related deaths (12.19%) during the same period. From 2023 to 2030, comorbid T2DM will result in 8.69% and 8.95% excess cases of severe HBV complications and liver-related deaths, respectively.

**Table 2:** Estimated excess incident cases of severe HBV complications caused by comorbid T2DM among the HBV-infected population

HBV complications	2006–2022 (thousands)				2023–2030 (thousands)			
	HBV-model	HBV-T2DM model	Absolute difference	Relative difference (%)	HBV-model	HBV-T2DM model	Absolute difference	Relative difference (%)
*Total population*								
Compensated cirrhosis	6932	7723	791	11.41	3456	3884	428	12.38
Decompensated cirrhosis	2632	2876	244	9.27	1243	1339	96	7.72
HCC	4293	4670	377	8.78	2415	2509	94	3.89
Total severe HBV complications	13,857	15,269	1412	10.19	7114	7732	618	8.69
Liver-related death	6529	7325	796	12.19	4035	4396	361	8.95
*Adults aged 60 years and older at baseline*								
Compensated cirrhosis	562	705	143	25.44	180	223	43	23.89
Decompensated cirrhosis	262	305	43	16.41	91	108	17	18.68
HCC	449	539	90	20.04	148	175	27	18.24
Total severe HBV complications	1273	1549	276	21.68	419	506	87	20.76
Liver-related death	683	879	196	28.70	284	336	52	18.31
*Adults between the ages of 45 and 59 at baseline*								
Compensated cirrhosis	1045	1236	191	18.28	392	444	52	13.27
Decompensated cirrhosis	485	564	79	16.29	165	181	16	9.70
HCC	896	976	80	8.93	296	331	35	11.82
Total severe HBV complications	2426	2776	350	14.43	853	956	103	12.08
Liver-related death	1344	1548	204	15.18	599	707	108	18.03

*T2DM* Type 2 diabetes mellitus, *HBV* Hepatitis B virus, *HCC* Hepatocellular carcinoma

The subgroup analysis results are presented in Table 2. For individuals aged 60 and older at baseline, comorbid T2DM led to 21.68% and 28.70% increases in severe HBV complications and liver-related deaths, respectively, from 2006 to 2022. Moreover, it is projected to cause 20.76% and 18.31% excess cases from 2023 to 2030 among this population. For individuals aged 45 to 59 at baseline, comorbid T2DM led to 14.43% and 15.18% excess cases of severe HBV complications and liver-related deaths, respectively, between 2006 and 2022. Furthermore, it is projected to result in 12.08% and 18.03% excess cases between 2023 and 2030 among this population.

### Validation and sensitivity analysis

The total number of HBV infections and CHB patients in 2016 projected by our models matches the figures reported by the China CDC. Additionally, the annual incident cases of HCC predicted in our models are comparable to those reported by the China National Cancer Center. The validation results of our models are presented in Additional file 1: Table S5.

The sensitivity analysis showed that 76.4% of the estimated excess cases of compensated cirrhosis, decompensated cirrhosis, HCC, and liver-related death due to comorbid T2DM from 2006 to 2022 varied within a range of 20%, while 89.6% varied within a range of 30%. The ranges of excess cases and deaths caused by comorbid T2DM among HBV-infected individuals were as follows: 4.63% to 17.41% for compensated cirrhosis, 4.87% to 18.47% for decompensated cirrhosis, 4.83% to 20.11% for HCC, and 9.09% to 20.88% for liver-related deaths (Additional file 1: Figs. S1–S4).

## Discussion

In this study, we found pronounced increasing trends in the incidence and number of patients affected by severe HBV complications from 2006 to 2030. Furthermore, we estimated that comorbid T2DM has caused approximately 10% excess cases of severe HBV complications or liver-related death from 2006 to 2022 among HBV-infected individuals in China. Given the current situation, we estimated a further 10% increase in these burdens among Chinese HBV-infected individuals over the next seven years due to comorbid T2DM. For HBV-infected individuals aged 60 and older, the excess disease burden caused by comorbid T2DM is above 20%.

We estimated a decreasing trend in the total number of HBV patients, while there was an increasing trend in the incidence and number of severe HBV complications among HBV-infected individuals from 2006 to 2030. These findings are consistent with previous modeling studies on HBV in China [27, 28]. The decline in the total number of HBV patients in China can be attributed to substantial progress in HBV control over the past few decades, including the expansion of HBV vaccination and prevention of mother-to-child transmission [36]. By 2017, the coverage of the birth dose of the HepB had reached 96%, and the prevalence of HBV among children aged 5 years had decreased to less than 0.5% in China [37]. However, despite these achievements, only one in ten individuals who require treatment are currently receiving it, and the annual rate of HBsAg seroclearance is less than 2% [32, 38]. Moreover, the rate of achieving functional cure for chronic HBV using currently available antiviral therapy is exceedingly low, at less than 1% [39]. Consequently, the number of HBV-infected individuals experiencing severe complications continues to rise, irrespective of the decrease in new infections. These results underscore the importance of enhancing the treatment and management of HBV-infected individuals to prevent the development of severe HBV complications and ultimately reduce the overall disease burden caused by HBV infection.

The incidence and cumulative cases of compensated cirrhosis, decompensated cirrhosis, HCC, and liver-related death were found to be higher in the HBV-T2DM model than in the HBV model. This can be attributed to the accelerated progression of liver disease resulting from comorbid T2DM among HBV-infected individuals. A systematic review of twenty epidemiological studies has previously indicated an association between T2DM and the accelerated progression of severe liver diseases in HBV-infected adults, including cirrhosis, HCC, liver transplantation, and liver-related death [17]. Mechanistic studies have provided possible biomedical explanations for this phenomenon. T2DM contributes to increased insulin resistance, fatty acid β-oxidation, and hepatic oxidative stress, which are risk factors for nonalcoholic steatohepatitis and ultimately contribute to the development of fibrosis and cirrhosis [40, 41]. T2DM is also associated with elevated levels of plasma free fatty acids [42], which accumulate and cause oxidative stress and inflammation, further increasing the risk of HCC in the presence of HBV infection [18, 19]. Furthermore, hyperglycemia and hyperinsulinemia have been identified as significant factors in the progression of liver fibrosis and HCC [20]. The prevalence of comorbid T2DM is expected to increase as the HBV-infected population ages. Future clinical research is needed to develop tailored treatment strategies targeting individuals with HBV-T2DM comorbidity to slow disease progression and enhance their quality of life.

Our study revealed that comorbid T2DM contributed to approximately 10% of additional cases of cirrhosis, HCC, and liver-related death among individuals with HBV infection in China from 2006 to 2022. Furthermore, our model projected a further 10% increase in these burdens over the next seven years due to comorbid T2DM. These results are attributed to the high disease burden of T2DM in China and the accelerated disease progression of HBV caused by comorbid T2DM [17, 26]. Our study revealed that comorbid T2DM accounted for approximately 20% of the excess severe HBV complications in individuals aged 60 and older with HBV infection. This result is primarily due to the higher prevalence of T2DM among older adults. The Chinese HBV-infected population is rapidly aging, with comorbid T2DM potentially becoming a major factor affecting the disease burden in this population [9]. Our findings indicate the importance of developing and implementing comprehensive strategies to alleviate the disease burden caused by comorbid T2DM among individuals infected with HBV. For instance, implementing screenings and strengthening treatment to improve the diagnosis, treatment, and control rates of T2DM among HBV-infected individuals. However, evaluating T2DM prevention and treatment policies among the HBV-infected population remains challenging under conditions where the existing situation is unclear. Modeling studies are needed to comprehensively evaluate these intervention strategies and health policies by conducting cost-effectiveness analyses. Our study can lay the groundwork for future research in this field.

The strength of our study lies in the incorporation of comorbid chronic diseases into traditional HBV models, allowing us to estimate the impact of these comorbidities on the disease burden of HBV. This approach provides valuable insights for future modeling studies on HBV comorbidities. However, our study has several limitations. First, the disease progression of HBV infection is a complex process, particularly for individuals with comorbid chronic diseases. Our models only include essential steps and outcomes in disease progression among patients with HBV-T2DM comorbidity. Second, we did not include sociocultural factors that influence the disease burden among HBV-infected individuals, such as educational level, racial group, and health behavior, due to the unavailability of relevant epidemiological data. Further studies are warranted to incorporate these factors into models when detailed data become available. Third, due to the scarcity of longitudinal research focusing on patients with HBV-T2DM comorbidity, we were unable to directly obtain parameters for disease progression in this population. Large-scale observational cohorts of patients with HBV-T2DM comorbidity are needed in the future to obtain more accurate parameters. Fourth, we did not incorporate regional differences in the epidemic situations of HBV and T2DM into our models due to the unavailability of data. Further studies are warranted to explore the disease burden at subnational levels. Lastly, our models did not incorporate the different severity levels and treatment states of T2DM due to the lack of relevant epidemiological parameters. The impact of T2DM on the disease progression of HBV infection may vary across different severity levels and treatment states of T2DM, further studies are needed to investigate the disease burden caused by T2DM with various severity and treatment states.

## Conclusions

The health status of the existing HBV-infected individuals in China is progressively deteriorating, which is further exacerbated by the presence of comorbid T2DM. Comorbid T2DM has caused approximately 10% additional cases of severe HBV complications and liver-related death among HBV-infected individuals in China between 2006 and 2022, with a projected further increase of 10% over the next seven years. Health policymakers need to develop and implement integrated management strategies for both HBV and T2DM to address the growing needs of HBV-infected individuals with complex health conditions.

### Supplementary Information

**Additional file 1: Table S1.** Annual transition probabilities for individuals infected with HBV alone. **Table S2.** Annual transition probabilities for individuals with both HBV infection and T2DM. **Table S3.** Distribution of disease states in the baseline population. **Table S4.** Prevalence of T2DM in the population infected with HBV. **Table S5.** Comparison of model results and published data. **Figure S1.** Estimated excess burden of compensated cirrhosis caused by comorbid T2DM among the HBV-infected population with varied parameters. **Figure S2.** Estimated excess burden of decompensated cirrhosis caused by comorbid T2DM among the HBV-infected population with varied parameters. **Figure S3.** Estimated excess burden of HCC caused by comorbid T2DM among the HBV-infected population with varied parameters. **Figure S4.** Estimated excess burden of liver-related death caused by comorbid T2DM among the HBV-infected population with varied parameters.

## Data Availability

The data utilized in this study are accessible through the links to public data resources outlined in the Methods section or can be obtained from the corresponding author on reasonable request.

## Abbreviations

CHB: Chronic hepatitis B
China CDC: The Chinese Center for Disease Control and Prevention
CI: Confidence interval
HBV: Hepatitis B virus
HBsAg: Hepatitis B surface antigen
HCC: Hepatocellular carcinoma
HepB: Hepatitis B vaccine
HR: Hazard ratio
NCDs: Noncommunicable diseases
RR: Relative risk
T2DM: Type 2 diabetes mellitus
WHO: World Health Organization
