# Supplementary material for: Assessing the impact of comorbid type 2 diabetes mellitus on the disease burden of chronic hepatitis B virus infection and its complications in China from 2006 to 2030: a modeling study
Source: Glob Health Res Policy. 2024 Jan 22;9:5. doi: 10.1186/s41256-024-00345-2 (PMC10801935; doi:10.1186/s41256-024-00345-2)
Supplement: Supplementary file 1 — Additional file 1: Table S1. Annual transition probabilities for individuals infected with HBV alone. Table S2. Annual transition probabilities for individuals with both HBV infection and T2DM. Table S3. Distribution of disease states in the baseline population. Table S4. Prevalence of T2DM in the population infected with HBV. Table S5. Comparison of model results and published data. Figure S1. Estimated excess burden of compensated cirrhosis caused by comorbid T2DM among the HBV-infected population with varied parameters. Figure S2. Estimated excess burden of decompensated cirrhosis caused by comorbid T2DM among the HBV-infected population with varied parameters. Figure S3. Estimated excess burden of HCC caused by comorbid T2DM among the HBV-infected population with varied parameters. Figure S4. Estimated excess burden of liver-related death caused by comorbid T2DM among the HBV-infected population with varied parameters. [file 41256_2024_345_MOESM1_ESM.docx]

**Assessing the impact of comorbid type 2 diabetes mellitus on the disease burden of chronic hepatitis B virus infection and its complications in China from 2006 to 2030: a modeling study**

**Additional File**

[**Supplementary Methods** 2](#_Toc149430682)

[**Table S1**. Annual transition probabilities for individuals infected with HBV alone 3](#_Toc149430683)

[**Table S2**. Annual transition probabilities for individuals with both HBV infection and T2DM 4](#_Toc149430684)

[**Table S3**. Distribution of disease states in the baseline population 5](#_Toc149430685)

[**Table S4**. Prevalence of T2DM in the population infected with HBV 6](#_Toc149430686)

[**Table S5**. Comparison of model results and published data 7](#_Toc149430687)

[**Figure S1**. Estimated excess burden of compensated cirrhosis caused by comorbid T2DM among the HBV-infected population with varied parameters. CHB, chronic hepatitis B; HBV, hepatitis B virus; T2DM, type 2 diabetes mellitus; HCC, hepatocellular carcinoma. 8](#_Toc149430688)

[**Figure S2**. Estimated excess burden of decompensated cirrhosis caused by comorbid T2DM among the HBV-infected population with varied parameters. CHB, chronic hepatitis B; HBV, hepatitis B virus; T2DM, type 2 diabetes mellitus; HCC, hepatocellular carcinoma. 9](#_Toc149430689)

[**Figure S3**. Estimated excess burden of HCC caused by comorbid T2DM among the HBV-infected population with varied parameters. CHB, chronic hepatitis B; HBV, hepatitis B virus; T2DM, type 2 diabetes mellitus; HCC, hepatocellular carcinoma. 10](#_Toc149430690)

[**Figure S4**. Estimated excess burden of liver-related death caused by comorbid T2DM among the HBV-infected population with varied parameters. CHB, chronic hepatitis B; HBV, hepatitis B virus; T2DM, type 2 diabetes mellitus; HCC, hepatocellular carcinoma. 11](#_Toc149430691)

[**Reference** 12](#_Toc149430692)

# **Supplementary Methods**

The definitions of the health states in the Markov model for the disease progression of HBV infection are presented as follows.

(1) Seroclearance

Seroclearance is defined as the absence of serum hepatitis B surface antigen (HBsAg) on at least two occasions, with a gap of at least 6 months between them [1].

(2) Asymptomatic carriers

Asymptomatic carriers are defined as patients with a positive HBsAg test, normal levels of alanine transaminase (ALT ≤ 40 U/L), and no evident signs of liver inflammation, necrosis, or fibrosis [2].

(3) Chronic hepatitis B (CHB)

CHB patients are defined as patients with a positive HBsAg test, sustained or recurrent abnormal elevated levels of ALT (ALT > 40 U/L), and obvious inflammation or necrosis of the liver [2].

(4) Compensated cirrhosis

Cirrhosis is defined as testing positive for HBsAg, showing signs of liver cirrhosis and/or portal hypertension in imaging examinations, or having histological features of cirrhosis confirmed through a liver biopsy. Cirrhosis is classified into compensated and decompensated stages based on the occurrence of severe complications such as ascites, esophageal variceal bleeding, and hepatic encephalopathy [2]. Compensated cirrhosis is defined as the pathological or clinical diagnosis of cirrhosis without the occurrence of these severe complications [2].

(5) Decompensated cirrhosis

Decompensated cirrhosis is defined as the pathological or clinical diagnosis of cirrhosis with the occurrence of severe complications such as ascites, esophageal variceal bleeding, or hepatic encephalopathy [2].

(6) Hepatocellular carcinoma (HCC)

HCC patients are defined as individuals who tested positive for HBsAg and were diagnosed with HCC according to the primary liver cancer diagnosis and treatment guidelines of China. [3].

(7) Death

Death consists of two categories: liver-related death and background death. Liver-related death refers to mortality caused by complications directly associated with liver disease or damage, such as liver failure, cirrhosis, and HCC. Background death refers to mortality resulting from causes not directly linked to liver diseases [4].

# Table S1. Annual transition probabilities for individuals infected with HBV alone

| Disease states | Base case | Range | Reference |
| --- | --- | --- | --- |
| **Asymptomatic carriers** |  |  |  |
| to HBsAg seroclearance | 0.015 | 0.0077-0.0183 | [5, 6] |
| to CHB | Age 20-39: 0.0023  Age ≥40: 0.0054 | Age 20-39:0.00115-0.00345  Age ≥40: 0.0027-0.0081 | [7-9] |
| to compensated cirrhosis | 0.0007 | 0.000-0.002 | [6, 10] |
| to HCC | 0.0006 | 0.0003-0.0011 | [11, 12] |
| **CHB** |  |  |  |
| to compensated cirrhosis | 0.016 | 0.013-0.019 | [10] |
| to HCC | Age 20-39: 0.002  Age ≥40: 0.0061 | Age 20-39:0.001-0.003  Age ≥40:0.00305-0.00915 | [10, 13, 14] |
| **Treated CHB** |  |  |  |
| to HBsAg seroclearance | 0.045 | 0.040-0.050 | [15, 16] |
| to compensated cirrhosis | 0.002 | 0.001-0.002 | [7, 12, 17, 18] |
| to HCC | 0.002 | 0.001-0.002 | [19] |
| **Compensated cirrhosis** |  |  |  |
| to decompensated cirrhosis | 0.04 | 0.02-0.05 | [20] |
| to HCC | 0.034 | 0.01-0.10 | [7, 10, 21-23] |
| to liver-rated death | 0.031 | 0.031-0.038 | [10, 24] |
| **Treated Compensated cirrhosis** |  |  |  |
| to decompensated cirrhosis | 0.019 | 0.009-0.046 | [17, 25] |
| to HCC | 0.0106 | 0.00954-0.01166 | [26] |
| to liver-related death | 0.017 | 0.012-0.048 | [18, 27] |
| **Decompensated cirrhosis** |  |  |  |
| to HCC | 0.034 | 0.01-0.10 | [10, 21, 28, 29] |
| to liver-related death | 0.17 | 0.10-0.25 | [10, 21, 30, 31] |
| **Treated decompensated cirrhosis** |  |  |  |
| to HCC | 0.0106 | 0.00954-0.01166 | [26] |
| to liver-related death | 0.095 | 0.056-0.140 | [7, 12, 32] |
| **HCC** |  |  |  |
| to liver-related death | 0.45 | 0.22-0.70 | [22, 28, 31, 33] |
| **Treated HCC** |  |  |  |
| to liver-related death | 0.26 | 0.25-0.27 | [7, 12, 34] |

CHB, chronic hepatitis B; HBV, hepatitis B virus; HBsAg, hepatitis B surface antigen; HCC, hepatocellular carcinoma.

# Table S2. Annual transition probabilities for individuals with both HBV infection and T2DM

| Disease states | Relative risks | Base case | Range | Reference |
| --- | --- | --- | --- | --- |
| **Asymptomatic carriers** |  |  |  |  |
| to HBsAg seroclearance | -- | 0.015 | 0.0077-0.0183 | [5, 6] |
| to CHB | -- | Age 20-39: 0.0023  Age ≥40: 0.0054 | Age 20-39: 0.00115-0.00345  Age ≥40: 0.0027-0.0081 | [7-9] |
| to compensated cirrhosis | 2.015 (1.390, 2.910) | 0.0014105 | 0.000973-0.002037 | [35] |
| to HCC | 1.36 (1.23, 1.49) | 0.000816 | 0.000738-0.000894 | [36] |
| **CHB** |  |  |  |  |
| to compensated cirrhosis | 2.015 (1.390, 2.910) | 0.03224 | 0.02224-0.04656 | [35] |
| to HCC | 1.36 (1.23, 1.49) | Age 20-39: 0.00272  Age ≥40: 0.008296 | Age 20-39: 0.00246-0.00298  Age ≥40: 0.007503-0.009089 | [36] |
| **Treated CHB** |  |  |  |  |
| to HBsAg seroclearance | -- | 0.045 | 0.040-0.050 | [15, 16] |
| to compensated cirrhosis | 2.015 (1.390, 2.910) | 0.00403 | 0.00278-0.00582 | [35] |
| to HCC | 1.36 (1.23, 1.49) | 0.00272 | 0.00246-0.00298 | [36] |
| **Compensated cirrhosis** |  |  |  |  |
| to decompensated cirrhosis | 1.792 (1.190, 2.690) | 0.07168 | 0.0476-0.1076 | [35] |
| to HCC | 2.36 (1.14, 4.85) | 0.08024 | 0.03876-0.1649 | [37] |
| to liver-rated death | 2.26 (1.05, 4.86) | 0.07006 | 0.03255-0.15066 | [37] |
| **Treated Compensated cirrhosis** |  |  |  |  |
| to decompensated cirrhosis | 1.792 (1.190, 2.690) | 0.034048 | 0.02261-0.05111 | [35] |
| to HCC | 2.36 (1.14, 4.85) | 0.025016 | 0.012084-0.05141 | [37] |
| to liver-related death | 2.26 (1.05, 4.86) | 0.03842 | 0.01785-0.08262 | [37] |
| **Decompensated cirrhosis** |  |  |  |  |
| to HCC | 2.36 (1.14, 4.85) | 0.08024 | 0.03876-0.1649 | [37] |
| to liver-related death | 2.26 (1.05, 4.86) | 0.3842 | 0.1785-0.8262 | [37] |
| **Treated decompensated cirrhosis** |  |  |  |  |
| to HCC | 2.36 (1.14, 4.85) | 0.025016 | 0.012084-0.05141 | [37] |
| to liver-related death | 2.26 (1.05, 4.86) | 0.2147 | 0.09975-0.4617 | [37] |
| **HCC** |  |  |  |  |
| to liver-related death | 1.05 (1.00, 1.10) | 0.4725 | 0.450-0.459 | [38] |
| **Treated HCC** |  |  |  |  |
| to liver-related death | 1.30 (1.09, 1.54) | 0.338 | 0.2834-0.4004 | [38] |

CHB, chronic hepatitis B; HBV, hepatitis B virus; HBsAg, hepatitis B surface antigen; HCC, hepatocellular carcinoma; T2DM, type 2 diabetes mellitus.

# Table S3. Distribution of disease states in the baseline population

| Disease states | Proportion (%) | Reference |
| --- | --- | --- |
| Seroclearance | 0.00 | -- |
| Asymptomatic carriers | 69.37 | -- |
| Chronic hepatitis | 21.51 | [39] |
| Compensated cirrhosis | 8.01 | [40, 41] |
| Decompensated cirrhosis | 0.99 | [40, 41] |
| Hepatocellular carcinoma | 0.13 | [42] |
| Death | 0.00 | -- |

# Table S4. Prevalence of T2DM in the population infected with HBV

| Age | Prevalence (%) | 95% Confidence interval |
| --- | --- | --- |
| <18 | 0.0 | 0.0-0.0 |
| 18-29 | 6.7 | 5.3-8.1 |
| 30-39 | 9.2 | 8.4-10.1 |
| 40-49 | 14.1 | 13.2-15.0 |
| 50-59 | 21.5 | 20.6-22.5 |
| 60-69 | 26.3 | 25.0-27.5 |
| ≥70 | 27.5 | 25.4-29.7 |

HBV, hepatitis B virus; T2DM, type 2 diabetes mellitus.

# Table S5. Comparison of model results and published data

| Variables | Published data | Reference | HBV model | HBV-T2DM model |
| --- | --- | --- | --- | --- |
| Total cases of HBV infection |  |  |  |  |
| 2016 | 87000000 | [43] | 88717000 | 88461000 |
| Total cases of CHB patients |  |  |  |  |
| 2016 | 28000000 | [43] | 26886000 | 26442000 |
| Annual HCC incident cases ^a^ |  |  |  |  |
| 2013 | 289,920 | [44] | 247000 | 262000 |
| 2014 | 292,000 | [45] | 241000 | 252000 |
| 2015 | 372,880 | [46] | 262000 | 274000 |
| 2016 | 311,040 | [47] | 296000 | 299000 |
| 2020 | 328,030 | [48] | 295000 | 306000 |

CHB, chronic hepatitis B; HBV, hepatitis B virus; HCC, hepatocellular carcinoma; T2DM, type 2 diabetes mellitus.

a: Previous literature and experts in hepatology have indicated that approximately 80% of liver cancer patients in China are infected with HBV [49].


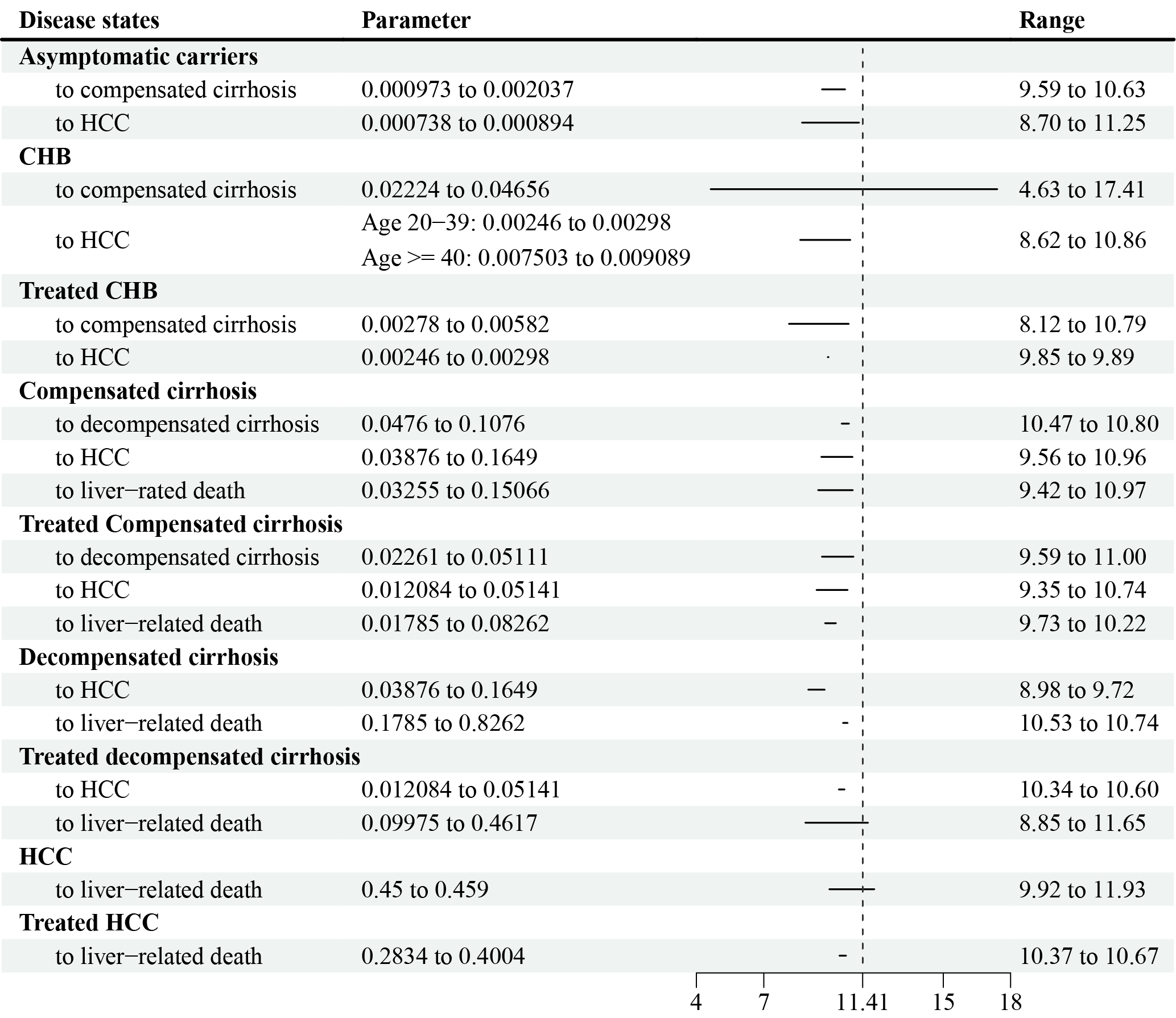


Figure S1. Estimated excess burden of compensated cirrhosis caused by comorbid T2DM among the HBV-infected population with varied parameters. CHB, chronic hepatitis B; HBV, hepatitis B virus; T2DM, type 2 diabetes mellitus; HCC, hepatocellular carcinoma.


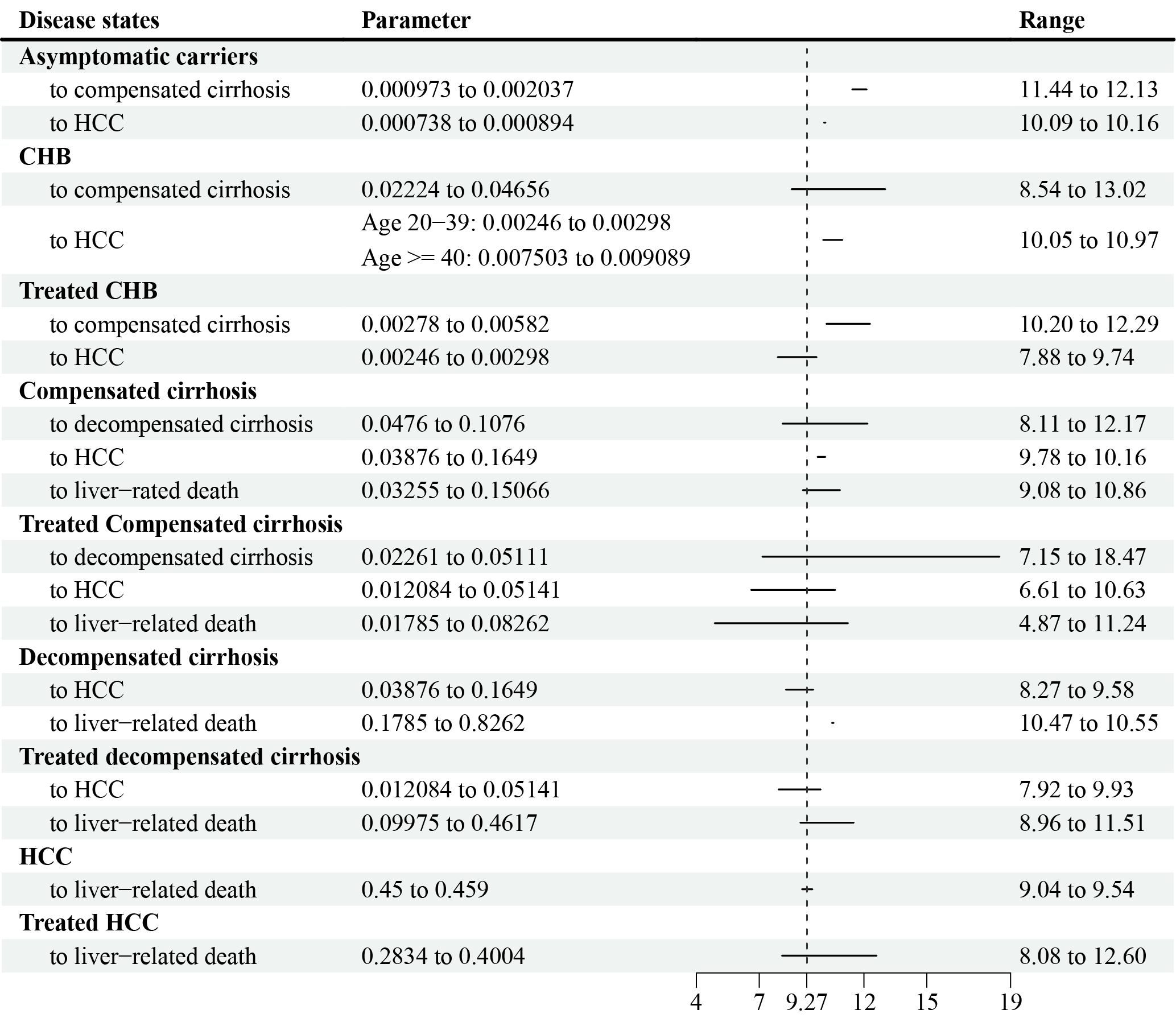


Figure S2. Estimated excess burden of decompensated cirrhosis caused by comorbid T2DM among the HBV-infected population with varied parameters. CHB, chronic hepatitis B; HBV, hepatitis B virus; T2DM, type 2 diabetes mellitus; HCC, hepatocellular carcinoma.
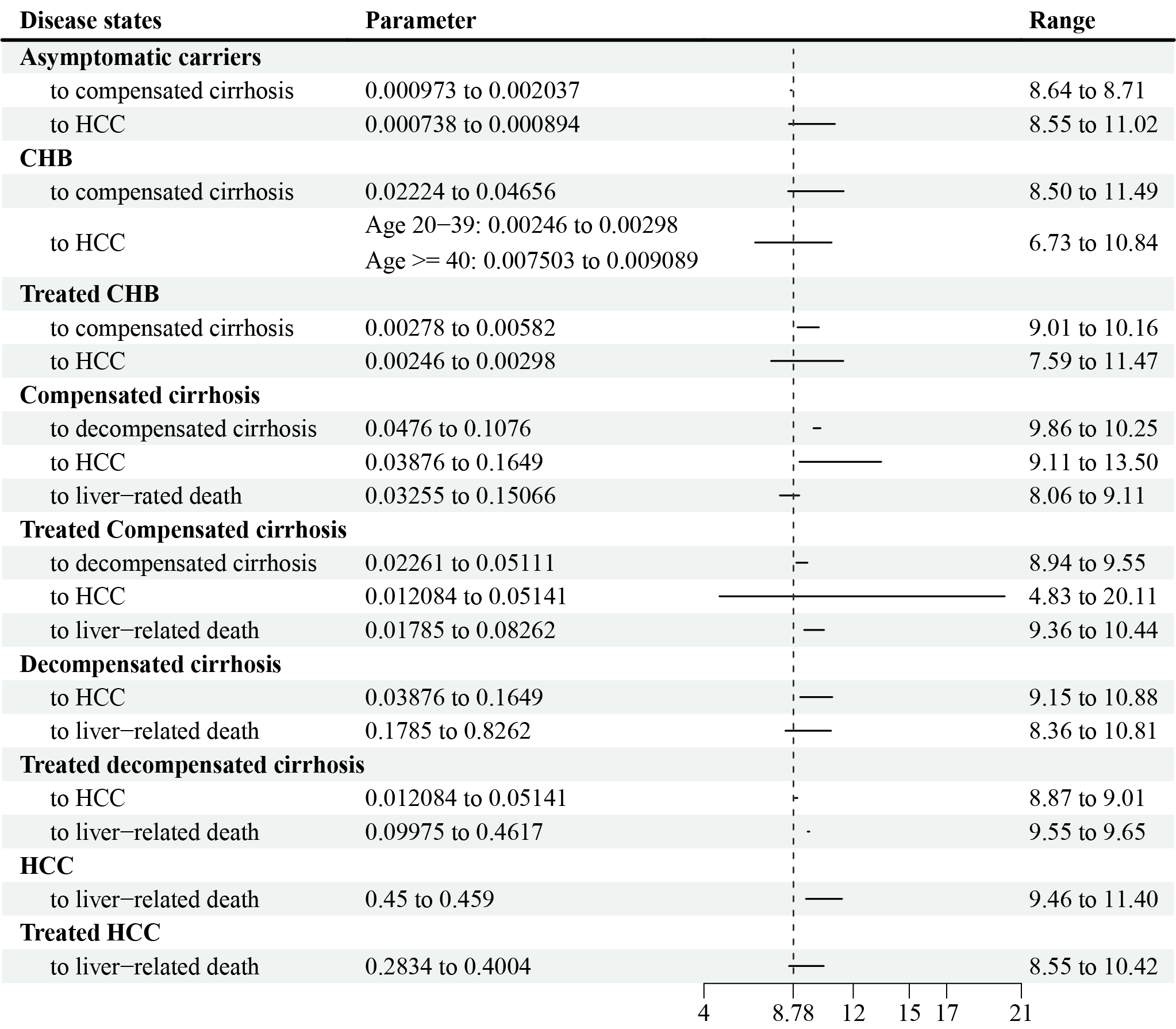


Figure S3. Estimated excess burden of HCC caused by comorbid T2DM among the HBV-infected population with varied parameters. CHB, chronic hepatitis B; HBV, hepatitis B virus; T2DM, type 2 diabetes mellitus; HCC, hepatocellular carcinoma.
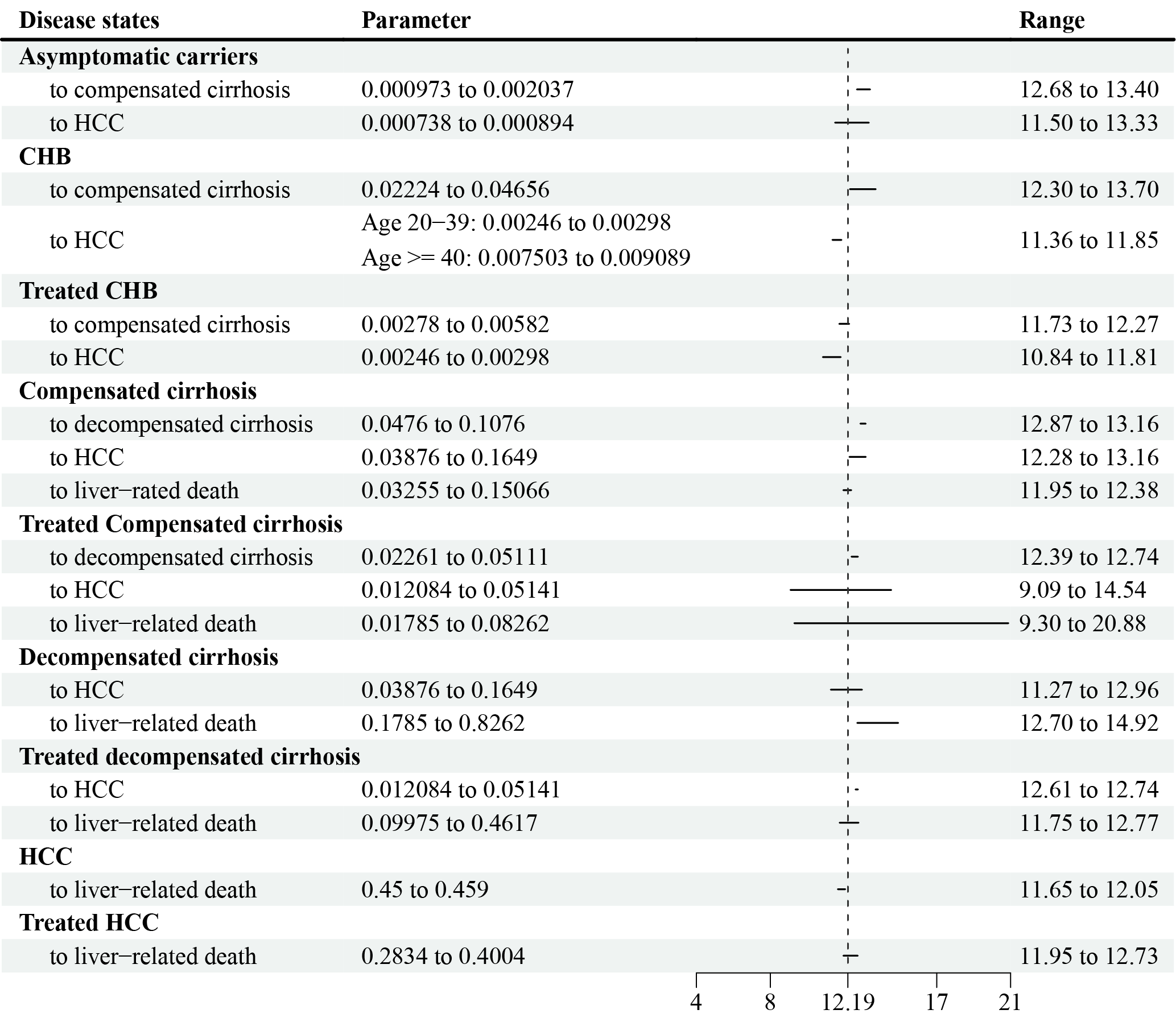


# Figure S4. Estimated excess burden of liver-related death caused by comorbid T2DM among the HBV-infected population with varied parameters. CHB, chronic hepatitis B; HBV, hepatitis B virus; T2DM, type 2 diabetes mellitus; HCC, hepatocellular carcinoma.

# **Reference**

1. Tout I, Loureiro D, Mansouri A, Soumelis V, Boyer N, Asselah T. Hepatitis B surface antigen seroclearance: Immune mechanisms, clinical impact, importance for drug development. J Hepatol. 2020;73(2):409-22.

2. Chinese Society of Hepatology and Chinese Medical Association. Guidelines for the prevention and treatment of chronic hepatitis B (version 2022). Chinese Journal of Clinical Infectious Diseases. 2022;15(6):401-26

3. National Health Commission of the People's Republic of China. Primary Liver Cancer Diagnosis and Treatment Guidelines (version 2022). Chinese Journal of Hepatology. 2022;30(4):367-88.

4. Toy M, Salomon JA, Jiang H, Gui H, Wang H, Wang J, et al. Population health impact and cost-effectiveness of monitoring inactive chronic hepatitis B and treating eligible patients in Shanghai, China. Hepatology. 2014;60(1):46-55.

5. Invernizzi F, Viganò M, Grossi G, Lampertico P. The prognosis and management of inactive HBV carriers. Liver Int. 2016;36 Suppl 1:100-4.

6. Zheng Y, Wu J, Ding C, Xu K, Yang S, Li L. Disease burden of chronic hepatitis B and complications in China from 2006 to 2050: an individual-based modeling study. Virol J. 2020;17(1):132.

7. Su S, Wong WCW, Zou Z, Cheng DD, Ong JJ, Chan P, et al. Cost-effectiveness of universal screening for chronic hepatitis B virus infection in China: an economic evaluation. Lancet Glob Health. 2022;10(2):e278-e87.

8. Hung HF, Chen TH. Probabilistic cost-effectiveness analysis of the long-term effect of universal hepatitis B vaccination: an experience from Taiwan with high hepatitis B virus infection and Hepatitis B e Antigen positive prevalence. Vaccine. 2009;27(48):6770-6.

9. La Torre G, Mannocci A, Saulle R, Colamesta V, Meggiolaro A, Mipatrini D, et al. Economic evaluation of HBV vaccination: A systematic review of recent publications (2000-2013). Hum Vaccin Immunother. 2016;12(9):2299-311.

10. Fattovich G, Bortolotti F, Donato F. Natural history of chronic hepatitis B: special emphasis on disease progression and prognostic factors. J Hepatol. 2008;48(2):335-52.

11. Chen JD, Yang HI, Iloeje UH, You SL, Lu SN, Wang LY, et al. Carriers of inactive hepatitis B virus are still at risk for hepatocellular carcinoma and liver-related death. Gastroenterology. 2010;138(5):1747-54.

12. Zhang L, Liu H, Zou Z, Su S, Ong JJ, Ji F, et al. Shared-care models are highly effective and cost-effective for managing chronic hepatitis B in China: reinterpreting the primary care and specialty divide. Lancet Reg Health West Pac. 2023;35:100737.

13. Tilson L, Thornton L, O'Flanagan D, Johnson H, Barry M. Cost effectiveness of hepatitis B vaccination strategies in Ireland: an economic evaluation. Eur J Public Health. 2008;18(3):275-82.

14. Wen WH, Chang MH, Hsu HY, Ni YH, Chen HL. The development of hepatocellular carcinoma among prospectively followed children with chronic hepatitis B virus infection. J Pediatr. 2004;144(3):397-9.

15. Terrault NA, Lok ASF, McMahon BJ, Chang KM, Hwang JP, Jonas MM, et al. Update on prevention, diagnosis, and treatment of chronic hepatitis B: AASLD 2018 hepatitis B guidance. Hepatology. 2018;67(4):1560-99.

16. Gish RG, Given BD, Lai CL, Locarnini SA, Lau JY, Lewis DL, et al. Chronic hepatitis B: Virology, natural history, current management and a glimpse at future opportunities. Antiviral Res. 2015;121:47-58.

17. Veldhuijzen IK, Toy M, Hahné SJ, De Wit GA, Schalm SW, de Man RA, et al. Screening and early treatment of migrants for chronic hepatitis B virus infection is cost-effective. Gastroenterology. 2010;138(2):522-30.

18. Chang TT, Gish RG, de Man R, Gadano A, Sollano J, Chao YC, et al. A comparison of entecavir and lamivudine for HBeAg-positive chronic hepatitis B. N Engl J Med. 2006;354(10):1001-10.

19. Wu CY, Lin JT, Ho HJ, Su CW, Lee TY, Wang SY, et al. Association of nucleos(t)ide analogue therapy with reduced risk of hepatocellular carcinoma in patients with chronic hepatitis B: a nationwide cohort study. Gastroenterology. 2014;147(1):143-51.e5.

20. Peng CY, Chien RN, Liaw YF. Hepatitis B virus-related decompensated liver cirrhosis: benefits of antiviral therapy. J Hepatol. 2012;57(2):442-50.

21. Wu B, Li T, Chen H, Shen J. Cost-effectiveness of nucleoside analog therapy for hepatitis B in China: a Markov analysis. Value Health. 2010;13(5):592-600.

22. Thiele M, Gluud LL, Fialla AD, Dahl EK, Krag A. Large variations in risk of hepatocellular carcinoma and mortality in treatment naïve hepatitis B patients: systematic review with meta-analyses. PLoS One. 2014;9(9):e107177.

23. Elgouhari HM, Abu-Rajab Tamimi TI, Carey WD. Hepatitis B virus infection: understanding its epidemiology, course, and diagnosis. Cleve Clin J Med. 2008;75(12):881-9.

24. Fattovich G, Pantalena M, Zagni I, Realdi G, Schalm SW, Christensen E. Effect of hepatitis B and C virus infections on the natural history of compensated cirrhosis: a cohort study of 297 patients. Am J Gastroenterol. 2002;97(11):2886-95.

25. Xu Y, Zhang YG, Wang X, Qi WQ, Qin SY, Liu ZH, et al. Long-term antiviral efficacy of entecavir and liver histology improvement in Chinese patients with hepatitis B virus-related cirrhosis. World J Gastroenterol. 2015;21(25):7869-76.

26. Choi J, Kim HJ, Lee J, Cho S, Ko MJ, Lim YS. Risk of Hepatocellular Carcinoma in Patients Treated With Entecavir vs Tenofovir for Chronic Hepatitis B: A Korean Nationwide Cohort Study. JAMA Oncol. 2019;5(1):30-6.

27. Lai CL, Shouval D, Lok AS, Chang TT, Cheinquer H, Goodman Z, et al. Entecavir versus lamivudine for patients with HBeAg-negative chronic hepatitis B. N Engl J Med. 2006;354(10):1011-20.

28. Siddiqui MR, Gay N, Edmunds WJ, Ramsay M. Economic evaluation of infant and adolescent hepatitis B vaccination in the UK. Vaccine. 2011;29(3):466-75.

29. Liaw YF. Natural history of chronic hepatitis B virus infection and long-term outcome under treatment. Liver Int. 2009;29 Suppl 1:100-7.

30. Keeffe EB, Dieterich DT, Han SH, Jacobson IM, Martin P, Schiff ER, et al. A treatment algorithm for the management of chronic hepatitis B virus infection in the United States: 2008 update. Clin Gastroenterol Hepatol. 2008;6(12):1315-41; quiz 286.

31. Nguyen VT, Law MG, Dore GJ. Hepatitis B-related hepatocellular carcinoma: epidemiological characteristics and disease burden. J Viral Hepat. 2009;16(7):453-63.

32. Tsai TY, Hung TH, Livneh H, Lin IH, Lu MC, Yeh CC. Chinese herbal medicine therapy and the risk of mortality for chronic hepatitis B patients with concurrent liver cirrhosis: a nationwide population-based cohort study. Oncotarget. 2018;9(26):18214-23.

33. Shepherd J, Jones J, Takeda A, Davidson P, Price A. Adefovir dipivoxil and pegylated interferon alfa-2a for the treatment of chronic hepatitis B: a systematic review and economic evaluation. Health Technol Assess. 2006;10(28):iii-iv, xi-xiv, 1-183.

34. Sun HC, Xie L, Yang XR, Li W, Yu J, Zhu XD, et al. Shanghai Score: A Prognostic and Adjuvant Treatment-evaluating System Constructed for Chinese Patients with Hepatocellular Carcinoma after Curative Resection. Chin Med J (Engl). 2017;130(22):2650-60.

35. Huang YW, Wang TC, Lin SC, Chang HY, Chen DS, Hu JT, et al. Increased risk of cirrhosis and its decompensation in chronic hepatitis B patients with newly diagnosed diabetes: a nationwide cohort study. Clin Infect Dis. 2013;57(12):1695-702.

36. Campbell C, Wang T, McNaughton AL, Barnes E, Matthews PC. Risk factors for the development of hepatocellular carcinoma (HCC) in chronic hepatitis B virus (HBV) infection: a systematic review and meta-analysis. J Viral Hepat. 2021;28(3):493-507.

37. Hsiang JC, Gane EJ, Bai WW, Gerred SJ. Type 2 diabetes: a risk factor for liver mortality and complications in hepatitis B cirrhosis patients. J Gastroenterol Hepatol. 2015;30(3):591-9.

38. Mrzljak A, Cigrovski Berkovic M, Giovanardi F, Lai Q. The prognostic role of diabetes mellitus type 2 in the setting of hepatocellular carcinoma: a systematic review and meta-analysis. Croat Med J. 2022;63(2):176-86.

39. Lu FM, Li T, Liu S, Zhuang H. Epidemiology and prevention of hepatitis B virus infection in China. J Viral Hepat. 2010;17 Suppl 1:4-9.

40. Tan M, Bhadoria AS, Cui F, Tan A, Van Holten J, Easterbrook P, et al. Estimating the proportion of people with chronic hepatitis B virus infection eligible for hepatitis B antiviral treatment worldwide: a systematic review and meta-analysis. Lancet Gastroenterol Hepatol. 2021;6(2):106-19.

41. Fleming KM, Aithal GP, Card TR, West J. The rate of decompensation and clinical progression of disease in people with cirrhosis: a cohort study. Aliment Pharmacol Ther. 2010;32(11-12):1343-50.

42. Institute for Health Metrics and Evaluation. Global Burden of Disease Study 2019 (GBD 2019) Data Resources. Available from: https://ghdx.healthdata.org/gbd-2019. Accessed 22 Aug 2023.

43. World Health Organization. Hepatitis in China. Available from: https://www.who.int/china/health-topics/hepatitis. Accessed 22 Aug 2023.

44. Chen W, Zheng R, Zhang S, Zeng H, Xia C, Zuo T, et al. Cancer incidence and mortality in China, 2013. Cancer Lett. 2017;401:63-71.

45. Chen W, Sun K, Zheng R, Zeng H, Zhang S, Xia C, et al. Cancer incidence and mortality in China, 2014. Chin J Cancer Res. 2018;30(1):1-12.

46. Chen W, Zheng R, Baade PD, Zhang S, Zeng H, Bray F, et al. Cancer statistics in China, 2015. CA Cancer J Clin. 2016;66(2):115-32.

47. Zheng R, Zhang S, Zeng H, Wang S, Sun K, Chen R, et al. Cancer incidence and mortality in China, 2016. Journal of the National Cancer Center. 2022;2(1):1-9.

48. World Health Organization. Global Cancer Observatory. Available from: https://gco.iarc.fr/. Accessed 22 Aug 2023.

49. de Martel C, Maucort-Boulch D, Plummer M, Franceschi S. World-wide relative contribution of hepatitis B and C viruses in hepatocellular carcinoma. Hepatology. 2015;62(4):1190-200.
